# A programmable platform for photonic topological insulators

**DOI:** 10.1515/nanoph-2024-0577

**Published:** 2025-02-14

**Authors:** Stuart Love, Mohamad Hossein Idjadi, Farshid Ashtiani, Howard (Ho-Wai) Lee, Andrea Blanco-Redondo

**Affiliations:** Department of Physics & Astronomy, 8788 University of California, Irvine, CA 92697, USA; Eddleman Quantum Institute, University of California, Irvine, CA 92697, USA; Nokia Bell Labs, 600 Mountain Avenue, New Providence, NJ 07974, USA; CREOL, University of Central Florida, Orlando, FL 32816, USA

**Keywords:** topological photonics, silicon photonics, programmable photonics, tunable devices, ring resonator

## Abstract

In the past decade, the field of topological photonics has gained prominence exhibiting consequential effects in quantum information science, lasing, and large-scale integrated photonics. Many of these topological systems exhibit protected states, enabling robust travel along their edges without being affected by defects or disorder. Nonetheless, conventional topological structures often lack the flexibility for implementing different topological models and for tunability postfabrication. Here, we present a method to implement magnetic-like Hamiltonians supporting topologically protected edge modes on a general-purpose programmable silicon photonic mesh of interferometers. By reconfiguring the lattice onto a two-dimensional mesh of ring resonators with carefully tuned couplings, we show robust edge state transport even in the presence of manufacturing tolerance defects. We showcase the system’s reconfigurability by demonstrating topological insulator lattices of different sizes and shapes and introduce edge and bulk defects to underscore the robustness of the photonic edge states. Our study paves the way for the implementation of photonic topological insulators on general-purpose programmable photonics platforms.

## 1 Introduction

The emergence of topological phenomena in condensed matter physics has provided opportunities for the exploration of topologically nontrivial systems in diverse materials [1]. Following the discovery of the quantum Hall effect [2], the observation of topological behavior, and topologically protected edge states, in other 1-D [3], 2-D [4], and even 3-D [5], [6], [7] systems have been rigorously investigated. While most topological insulator studies are predominantly fermion-based, bosonic systems, such as photonic systems, have shown analogous topological behavior [8]. These topological photonic systems have found ramifications in non-Hermitian and nonlinear physics and applications in quantum information science lasers and communications [9].

Despite the tremendous progress in the field, most of the experimental platforms for photonic topological insulators implement a specific model and lack reconfigurability. Only with the recent introduction of programmable integrated photonics [10] into the topological photonics realm, has full reconfigurability of photonic topological insulators become a real prospect. Following initial demonstrations of reconfigurable light steering in topological modes [11], [12], [13], [14], [15], [16], earlier this year, we have witnessed the true advent of programmable topological photonics [17], [18], [19]. In particular, Ref. [18] demonstrated the use of a general-purpose, commercial, programmable integrated photonic platform to implement different topological Hamiltonians. Specifically, this work demonstrated the presence of zero-dimension topologically localized modes in the Su–Schrieffer–Heeger (SSH) model and in the breathing Kagome model. Although these results highlight the possibility of implementing different topological Hamiltonians in the same integrated photonics platform, a concrete path for implementation of a true photonic topological insulator in a general-purpose programmable platform is still lacking.

In this work, we outline a method to implement a two-dimensional quantum spin-Hall Hamiltonian in a reconfigurable mesh of Mach–Zehnder interferometers (MZIs). Our method is based on constructing loop paths for light forming ring resonators, and subsequently controlling the coupling strengths and phases between nonadjacent resonators by using connecting waveguides and phase-shifters. By using simulations with realistic parameters, we demonstrate the appearance of a topological band gap and the presence of topologically protected edge transport. We analyze the robustness of the topological edge transport against disorder in the coupling strengths and phases, and in the presence of large fabrication defects, like missing resonators. Further, we elucidate the impact of the lattice size on the topological transport properties. Our results highlight a concrete route to implement two-dimensional topological insulators on general-purpose integrated photonic platforms that are commercially available, and thus, we expect these findings will fast-track the development of photonic topological insulators and their applications.

## 2 Programmable topological silicon photonics design

**Figure 1:**
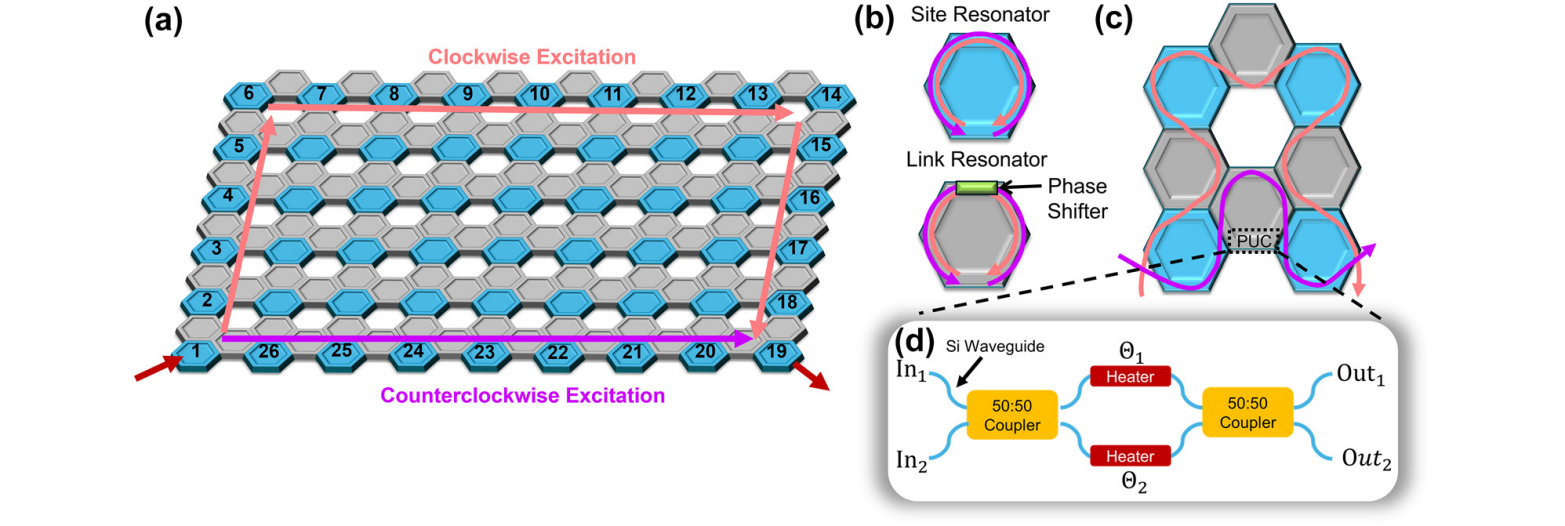
Overview of topological resonator lattice. (a) Overall view of the lattice created in the iPronics system. (b) The 6×9 lattice has two types of resonators: site and link. Since they are identical in size, to separate the resonant frequencies we attach a phase shifter on the link resonators, which shifts the resonant frequency by ½ FSR when set to a value of π. (c) A plaquette of resonators highlighting the paths taken either clockwise or counterclockwise. (d) Diagram of one of the PUCs imbedded in the iPronics simulation.

The general-purpose programmable silicon photonics platform simulated in this work is depicted in Figure 1a. It relies on a hexagonal mesh of silicon photonics MZIs. Each MZI forms a programmable unit cell (PUC), with two inputs and two outputs, as depicted in Figure 1d. By tuning the phase of the light going through each arm of the MZI (*θ*_1_, *θ*_2_), the power and phase of each output is controlled by the following 2x2 complex unitary transfer matrix [18], [20]:

$$T\left(\theta_{1},\theta_{2}\right)=i{\text{e}}^{\text{i}\phi }\left[\begin{array}{cc} \sin\left(\Delta \right) & \cos\left(\Delta \right)\\ \cos\left(\Delta \right) & -\sin\left(\Delta \right) \end{array}\right],$$

with

$$\phi =\frac{\theta_{1}+\theta_{2}}{2},$$

and

$$\Delta =\frac{\theta_{1}-\theta_{2}}{2}.$$

In this way, the light path in the mesh can be configured to form two-dimensional (2D) arrays of coupled resonators, with the hopping parameter between resonators given by

$$J=\frac{\text{si}{\text{n}}^{2}\left(\Delta \right)}{4}\text{FSR,}$$

where FSR is the resonator’s free spectral range. Further details on the transfer matrices and connection of PUCs are described in the Supplementary Notes 1 and 2.

We propose a specific 2D configuration of the programmable silicon photonics arrangement that implements a quantum-spin Hall Hamiltonian supporting a clockwise and a counterclockwise edge state, as originally proposed in Ref. [21] and implemented in a fixed array of microring resonators in Ref. [22]. In an analogy to this work, we create a mesh comprised of *site* resonators, which support clockwise (CW) and counterclockwise (CCW) resonant modes, and *link* resonators, which are off-resonance with the site resonators and serve to control the coupling phase between *site* resonators, as depicted in Figure 1b. However, while in Ref. [15], the hopping phase between resonators was determined by a positional shift of the link resonators; in our work, we can accurately control the hopping phase by programming one of the phase shifters in the link resonators. This misaligns the two resonant frequencies, which is a necessary step as both resonators are the same size and thus have the same natural resonances.

We used an iPronics custom simulation mesh [23] with nearly 700 PUCs to implement a lattice with 54 site and 93 link resonators. This simulation mesh is a larger version of the commercially available iPronics Smartlight Processor hardware, thus realistic. Each resonator is composed of six PUCs, where each PUC is ≈ 811 µm long, yielding an FSR of 115 *pm* (14.6 GHz). In all the simulated configurations, light is coupled into (out of) the system through the resonator located at the bottom left (right) of the lattice shown in Figure 1a. The direction of excitation of the input ring determines whether the CW or the CCW edge state is excited. The hopping parameter between rings is set to J = 2.9 GHz (see Supplementary Note 1), and the phase shifters in the link resonators are tuned provide a phase shift of π, offsetting the resonant frequency of the link resonators by ½ FSR with respect to the site rings.

The input power in all our simulations is set to 0 dBm, and the optical power in this lattice is monitored by tapping 1 % of the light of each edge *site* resonator (the optical power in the bulk *site* resonators is not accessible in our system).

## 3 Results

**Figure 2:**
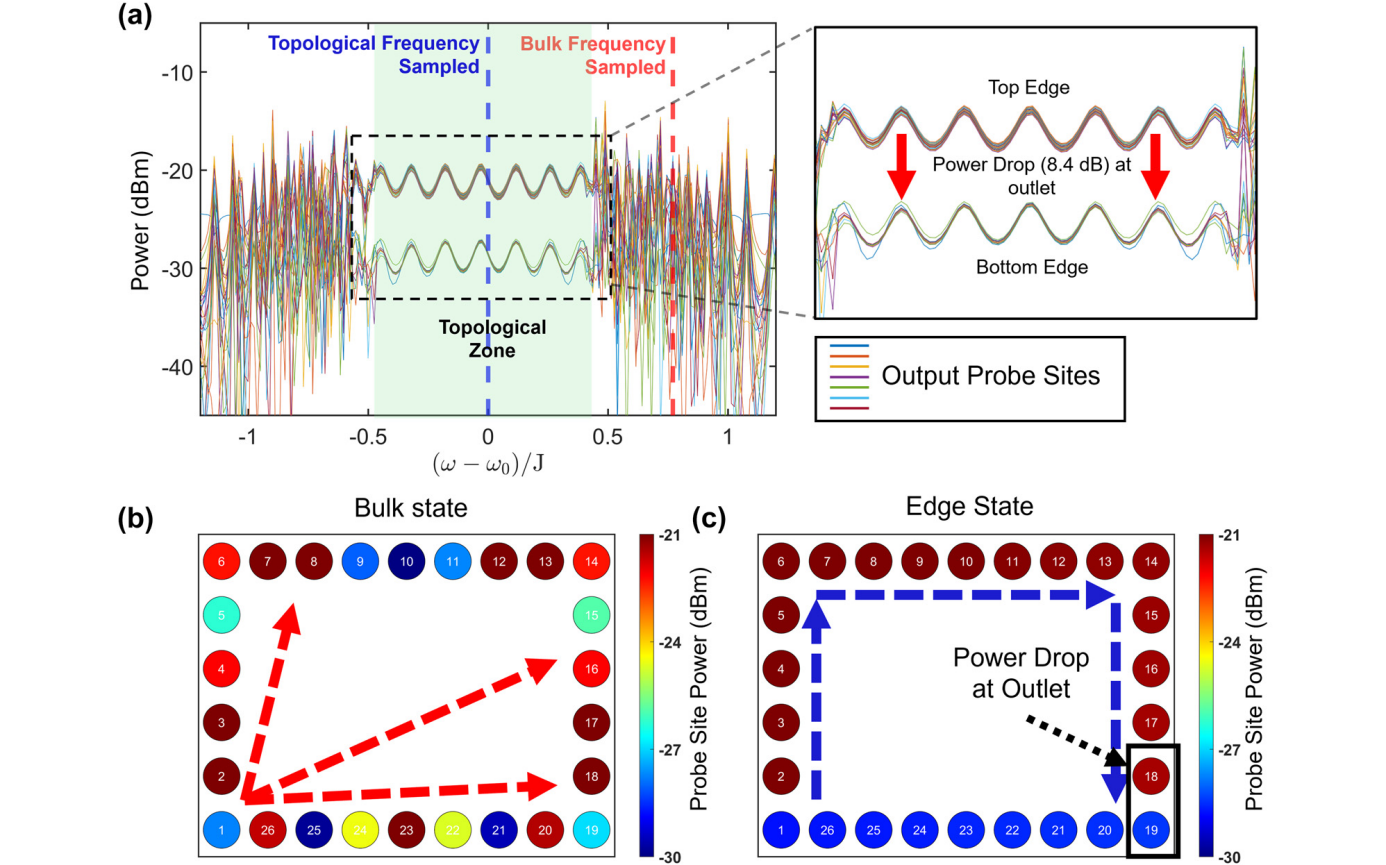
The excitation of the lattice from the bottom left resonator in the lossless case. (a) The spectra of all 26 ports from this configuration. The topological edge state of the same clockwise excitation, represented by the blue dashed line a). Inlet shows how the power, when in the topological edge state, will propagate around the edge of the lattice (clockwise in this iteration) and then mostly exit at the output port at site 19, thus dropping and forming the two distinct regions. (b) The bulk state of the lattice, frequency at the red dashed line in (a). (c) The topological state of the lattice, frequency at the blue dashed line in (a).

To probe the existence of topological features, we excite the structure at ring #1, at the bottom left corner of the lattice, and plot the monitored power in each of the 26 site resonators at the edge of the lattice as a function of the angular frequency (Figure 2a) normalized to the hopping parameter J and shifted to the center as highlighted by the blue line. The plotted spectra show a clear bandgap around the center frequency (*ω*_0_ = 2*π* × 193.569 THz) surrounded by two areas of continuum. The band gap (shaded green) holds two discrete levels that, as highlighted in the inset, correspond to the power measured in the rings that form part of the top path (as shown in Figure 1a as the orange path) and bottom edge residual power, respectively. To elucidate the origin of these features, we analyze the power distribution along the edge at two specific frequencies: one within the continuum (dashed red line) and one within the band gap (dashed blue line) (Figure 2b and c). Because the probe sites are sampling at a rate of 0.01, the output power reported here is low (<−20 dBm), but these values are consistent with the input power of 0dBm.

The power distribution along the edge of the frequency in the continuum is shown in Figure 2b. Despite initial excitation in a clockwise manner, the emitted power disperses through the lattice. Furthermore, as the light propagates away from the excitation point, its power fluctuates, indicating a lack of directionality. This suggests that this frequency propagates through the bulk.

In contrast, when the same excitation is applied at a frequency in the band gap, the power travels clockwise around the edge, maintaining a uniform power, as shown in Figure 2c. When the light gets to the output ring at the bottom right corner (ring #19), it predominately couples out. The remaining power continues propagating clockwise along the bottom edge, with a power drop of ∼ 8.4 dB. This explains the two separate levels in Figure 2a that differ exactly by this amount. The high power observed at the edges, in comparison with the bulk in Figure 2b, and its uniform distribution along the edge, suggests the excitation of the clockwise topological edge mode.

**Figure 3:**
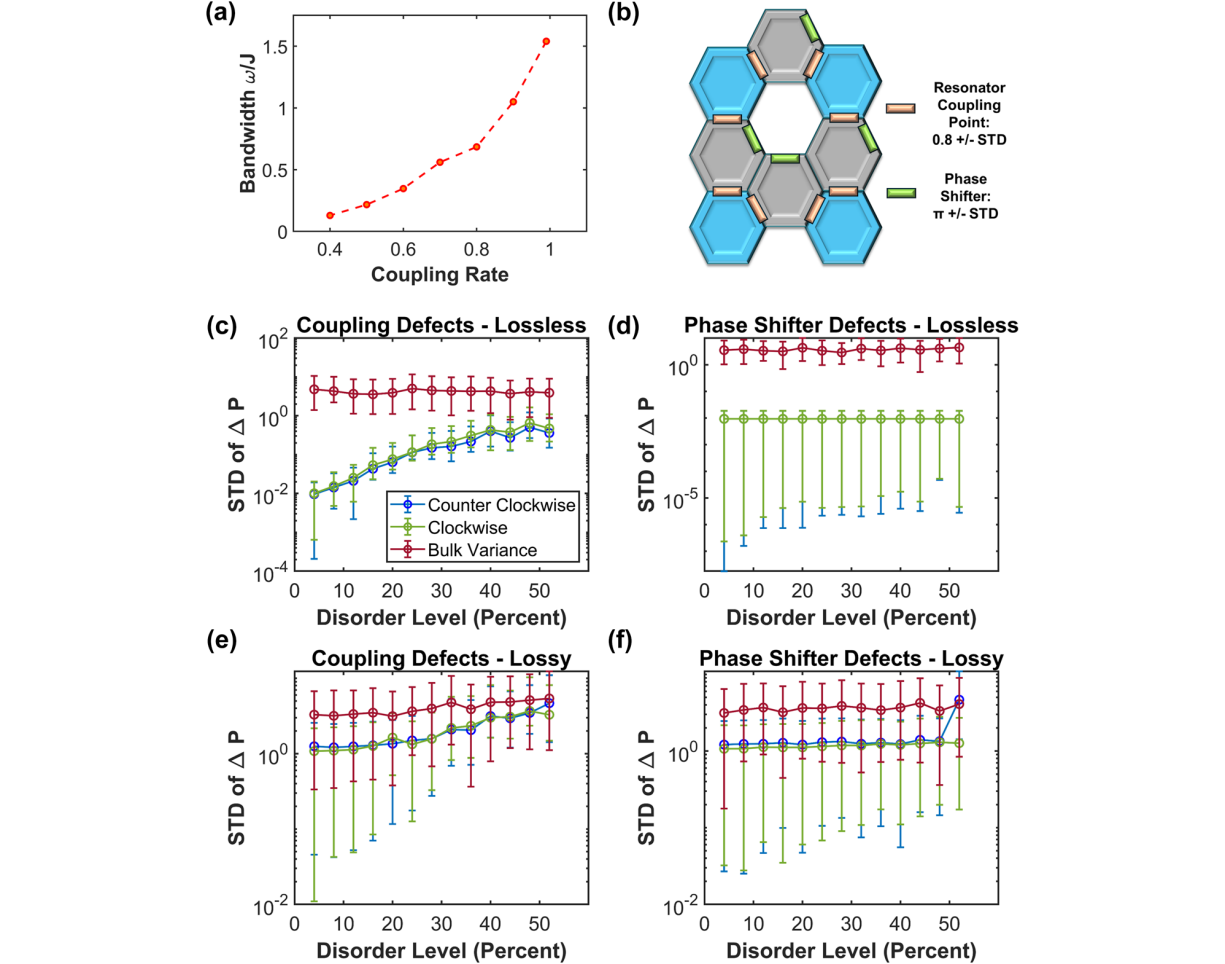
Bandwidth and manufacturing defect simulations. (a) Bandwidth of the topological state as a function of the coupling rate. (b) Sample plaquette highlighting how manufacturing disorder was introduced into the system. (c)–(f) Testing the tolerancing of the lattice structure by introducing standard deviations into the resonator coupling and the phase shifters. (c) Lossless case of coupling disorder, (d) lossy case of coupling disorder, (e) lossless case phase shifter disorder, and (f) lossy case of phase shifter disorder. Error bars shown are the 20th and 80th percentile of the 10 simulations run for each percent disorder.

The programmability of the platform allows us to tune the topological properties of the structure. For instance, the width of the topological band gap is plotted as a function of the coupling strength in Figure 3a. The bandwidth increases with the coupling rate, which can be explained as the broadening of the resonance in the over coupled regime. At a coupling rate of 1, the coupled resonators become a waveguide path, and the state is no longer topological. In Supplementary Note 3, the phase shifters are adjusted continuously and movement of the state through the FSR is shown.

To ascertain the efficacy of topological states compared to bulk states in preserving path coherence, the same power differentials between site n and site n+1 are used, that is, $\Delta P=P\left(n\right)-P\left(n+1\right)$ . This metric serves as an indicator of the propagation quality along the edge. Enhanced consistency in power differentials signifies the persistence of topological behavior within our state. That is, we take the power differentials and conduct a simple statistical analysis of the collected values and generate the standard deviations (STD). High levels of STD indicate that the state may no longer exhibit topological behavior. The employment of this power differential facilitates the visualization of the lattice’s resilience to disorder. Particularly, quantifying the variance of this dataset emerges as an effective metric for assessing the impact of disorder on the system. Further discussion of this metric is described in Supplementary Note 4 *.*

We then introduced random variations in both the coupling rates and the phase shifter values (Figure 3b–f) through a Gaussian distribution with the mean equaling the designed value and an increase STD. We start by looking at our original system with *J* = 2.9 GHz coupling rate and introduce a random level of variance in the coupling values. The levels of the random Gaussian variation in the coupling ratio were introduced as disorder levels in percent (2 × STD/mean x 100 = Percent Disorder). To measure the overall quality of the system after introducing disorder, we measured the difference in power between adjacent probe sites. In an ordered system, the variance in the power difference should be low. However, as the disorder begins to rise in the system so should the variance in the output powers, since the system begins to behave more randomly.

The topological behavior remains despite having this variance. Here, we are measuring the strength of our topological state by observing the power difference between adjacent sites. These values are collected the STD displayed on the *y* axis, with the level of disorder on the *x* axis. If we increase the disorder of coupling all the way to 52 %, there is a larger STD in the power difference from adjacent sites across the edge state and the topological state begins to encroach on the bulk level of variance; however, it remains robust despite such a strong amount of disorder introduced. In the lossy case (Figure 3e and f), the system can deal with up to 35–40 % disorder before the edge state breaks down. That is, the error bars of the CW and CCW states are now within the mean of the bulk state; thus, it becomes difficult to differentiate between the two states. However, if we introduce a variance in the phase shifter, we can still see that the system remains intact with little change to the power difference variance. The system can handle larger levels of disorder in the phase shifter, both in the lossless and lossy cases.

**Figure 4:**
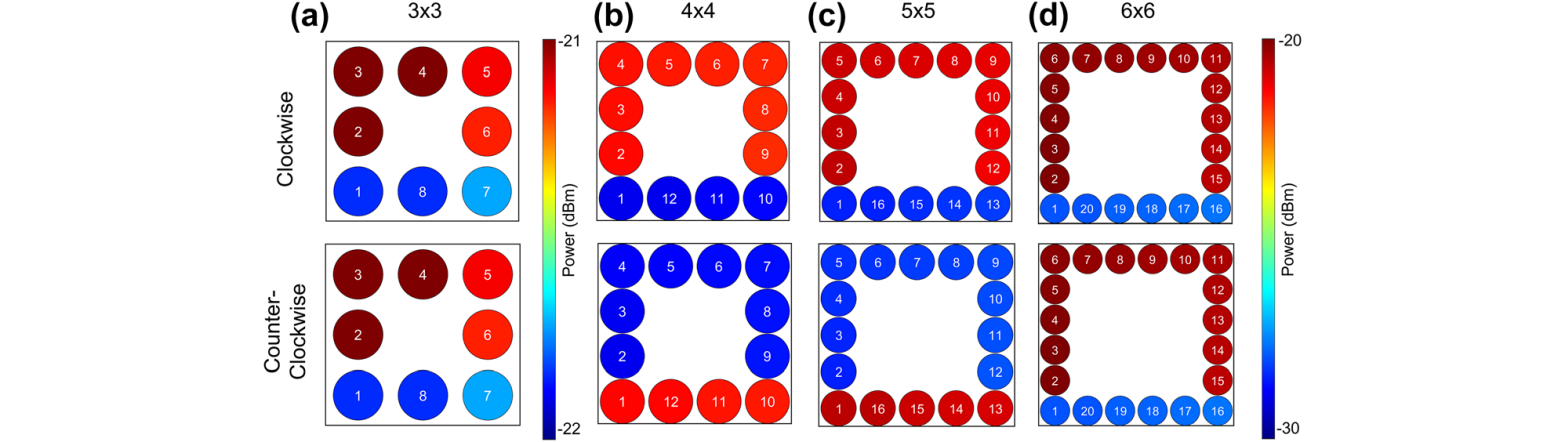
Resizing the configurations (a) 3×3, (b) 4×4, (c) 5×5, and (d) 6×6. In each of the figures, the clockwise and counterclockwise states are shown. Even in the smallest case of 3×3, we see signs of topological effects, but note that the power difference between the top edge and the bottom edge is significantly smaller than the other configurations. As the size of the lattice increases, the topological effects become more prominent and the spacing in between the upper (lower) and lower (upper) becomes larger.

To show another aspect of the reconfigurability of the system, we rearranged the size of the topological lattice to 3×3, 4×4, 5×5, and 6×6 (Figure 4). In the smallest limit, we observe that the 3×3 lattice exhibits edge states, despite the bulk being one site lattice. However, the difference in power between the top edge and bottom edge has been reduced to less than a 1 dB, as compared with ∼ 8 dB in the other configurations. When extended to the larger systems, the edge states become more prominent, showing that while the original lattice was designed for the 6×9 site size, the model created here can be modified to any arbitrary size or shape.

**Figure 5:**
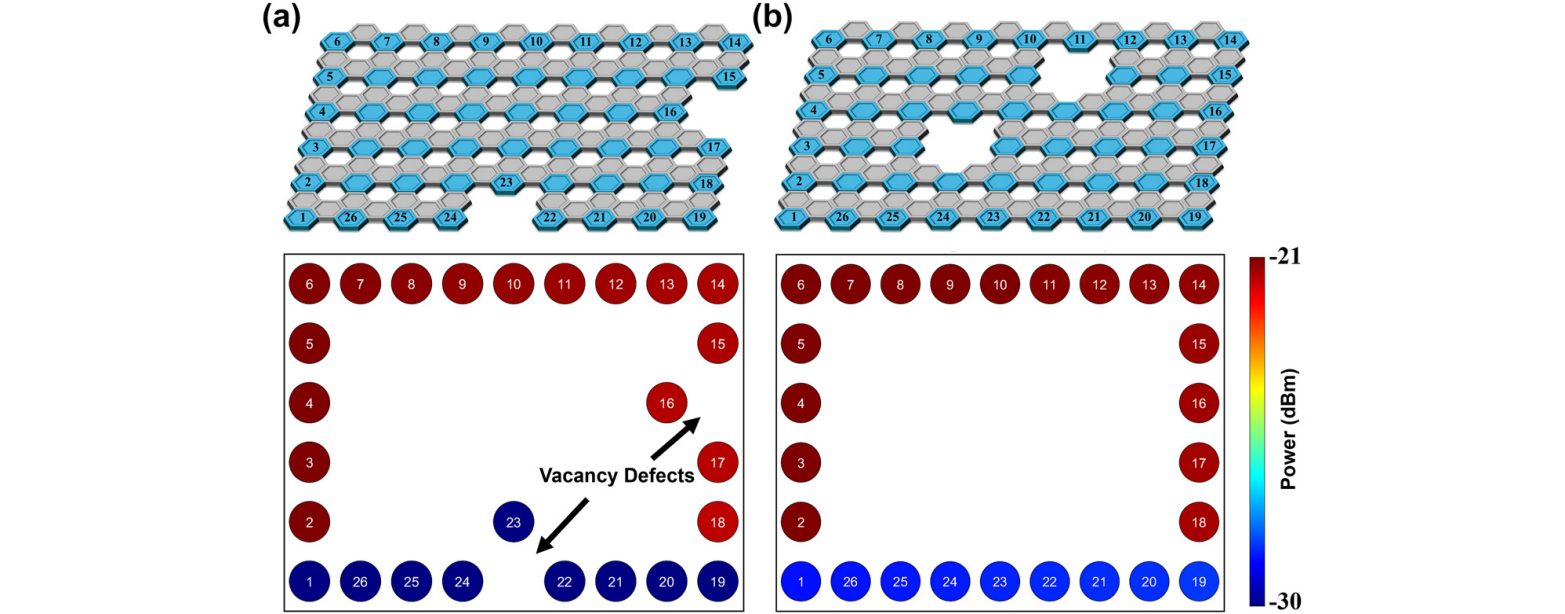
Removing plaquettes from the lattice to test the robustness of the topological effects. (a) Removing the edge sites of the lattice shows to have little effect on the edge state. (b) Removing the bulk lattice sites has no effect on the edge state.

The most stringent test of topological states is introducing defects along the edge path. If our system is a topological photonic insulator, the edge mode will go around the defect and continue without backscattering. To simulate this, plaquettes were removed (3 link resonators and a site resonator on the edge), and the power was directly monitored next to the removed plaquette. The results of removing two plaquettes (edge and bulk) are shown in Figure 5. While there is an increase in the power to the output port in the former case, both remain relatively unaffected by these defects. The power differential from adjacent sites remained consistent and the state maintained orderly power distributions as highlighted in the previous cases. This then demonstrated how the state moved around the defects rather than scattering in the opposite direction or into the bulk. With further development on highly tunable materials, this can be incorporated into a manufacturable structure to achieve high fidelity quantum transport waveguide or high efficiency topological optical modulator. As programmable integrated photonic platform progress toward lower loss and faster tuning speeds – with the incorporation of electro-optic materials like thin-film lithium niobate and barium titanium oxide – the method presented here could have an impact on topologically protected quantum information platforms [24], [25], [26], [27], [28].

## 4 Conclusions

We have proposed a method to implement a quantum-spin Hall topological insulator on a general-purpose programmable integrated photonics lattice. Using simulations we showed the presence of topologically protected edge modes and their robustness to different kinds of disorder and defects. The simulated topological modes show a 45 pm (5.8 GHz) bandwidth around wavelength of 1,550 nm, with full wavelength tunability from end to end of the resonator’s free spectral range. In the presence of disorder (i.e., random coupling and phase variation as well as defects), the edge states remain robust, as shown by the low variance in site-to-site losses, indicating our edge state is minimally impacted by changes in the system. Because of the system’s reconfigurability, topological lattices of any shape can be designed using this platform, highlighting the simulators effectiveness to show topological photonic effects. The simulator mesh is within technological capabilities of silicon photonics and can be fabricated with the current commercially available platforms.

## Supplementary Material

Supplementary Material Details
