# Supplementary material for: A programmable platform for photonic topological insulators
Source: Nanophotonics. 2025 Feb 14;14(3):367–73. doi: 10.1515/nanoph-2024-0577 (PMC11831389; doi:10.1515/nanoph-2024-0577)
Supplement: Supplementary file 1 — Supplementary Material Details [file j_nanoph-2024-0577_suppl_001.docx]

**Supplementary Information:**

**A programmable platform for photonic topological insulators**

Stuart Love^[[1]](#footnote-2)^,^2,3^ *, Mohamad Hossein Idjadi^[[2]](#footnote-3),3^ *, Farshid Ashtiani ^3^*, Howard (Ho-Wai) Lee1^,2^, Andrea Blanco-Redondo^4^

^1^ Department of Physics & Astronomy, University of California, Irvine, CA 92697, United States

^2^ Eddleman Quantum Institute, University of California, Irvine, CA 92697, USA

^3^ Nokia Bell Labs, 600 Mountain Avenue, New Providence, NJ 07974, United States

^4^ CREOL, University of Central Florida, Orlando, FL 32816

Corresponding authors: Howard (Ho-Wai) Lee ([hwlee524@uci.edu](mailto:hwlee524@uci.edu)), Andrea Blanco-Redondo ([Andrea@creol.ucf.edu](mailto:Andrea@creol.ucf.edu)), Stuart Love ([stlove@uci.edu](mailto:stlove@uci.edu))

* Indicates equal contributions

**Supplementary Note 1: Tight Binding Model and Plaquette Hamiltonian**

The basis for the topological Hamiltonian for resonator lattice presented in this work is shown below and has previously been used in similar literature [1] for a plaquette of link and site resonators (see Fig. 1 in main text):

$$H= -J[{\hat{a}_{1}}^{\dagger}\hat{a}_{2}e^{-i\phi_{12}}+{\hat{a}_{3}}^{\dagger}\hat{a}_{2}+{\hat{a}_{4}}^{\dagger}\hat{a}_{3}e^{-i\phi_{34}}+{\hat{a}_{1}}^{\dagger}\hat{a}_{4}]$$

The $\hat{a}^{\dagger}, \hat{a}$ respectively represent the creation and annihilation operators while $\phi_{ij}$ is the phase accumulation between sites *i* and *j.* We use a synthetic magnetic field achieved through the use of ring resonant structures This Hamiltonian is similar to other tight binding electronic and photonic systems under a magnetic field [2] but includes a few differences. Firstly, the size of the resonant structure is much larger than in previous works, thus reducing the Free Spectral Range (FSR). Secondly, past work has used 2D topological resonator lattices where the hopping phase is determined by the positional shift of the link resonator [1]. In this work we use phase shifters located on the link sites to induce this path change. And lastly, the work presented has dynamic tuning capabilities and reconfigurability, thus modifying these hopping parameters becomes possible.

**
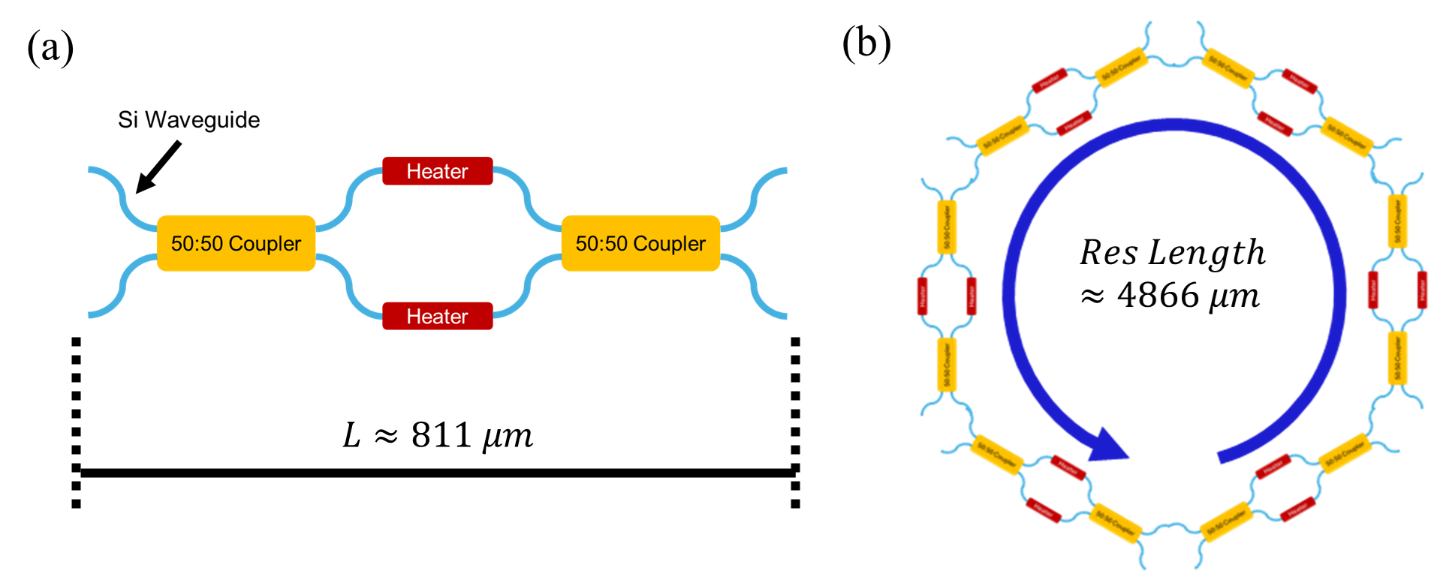
**

Figure S1: (a) A programable unit cell (PUC) that consists of a 50/50 couple and heater. These heaters are adjusted so that the light that comes in on the left can be sent in any ratio between 0 and 1 out of the top path. Whatever is remaining gets released down the other path. (b) By linking 6 PUC s together and sending the light through the inside path, a ring resonator can form by programming the individual PUCs to take the inside path, with one PUC as the input/output.

**Supplementary Note 2: Detailed Description of the MZI Programmable Unit Cell.**

Figure S1 provides detailed description of the device simulated. Each of the PUCs are connected through one the outputs in a hexagonal pattern. The 50/50 multimode interference (MMI) couplers split the input light, and the heaters (phase shifters) are adjusted such that it exits into a single path on the PUC. This is then iterated around the loop forming a ring resonator with the length shown in the figure. In the configuration described there is one entry and exit PUC that does not send all the light into the resonator (typically set to 0.8 in the main text). The number of entry and exit points varies depending on the resonator (4 one the bulk sites and 2-3 on the edge sites).

The power flow through the PUC can be calculated using Transfer Matrices representing the MMIs and phase shifters. Their matrices are represented as (and visually shown in figure S2):

$$T_{MMI}=\frac{1}{\sqrt{2}}\left[ \begin{matrix} 1 & i \\ i & 1 \end{matrix} \right]$$

$$T_{PS}=\left[ \begin{matrix} e^{i\theta_{1}} & 0 \\ 0 & e^{i\theta_{2}} \end{matrix} \right]$$

Which represent the MMI and Phase shifter respectively. Multiplying these out:

$$T=\frac{1}{2}\left[ \begin{matrix} 1 & i \\ i & 1 \end{matrix} \right]\left[ \begin{matrix} e^{i\theta_{1}} & 0 \\ 0 & e^{i\theta_{2}} \end{matrix} \right]\left[ \begin{matrix} 1 & i \\ i & 1 \end{matrix} \right]=\frac{1}{2}\left[ \begin{matrix} e^{i\theta_{1}}-e^{i\theta_{2}} & i(e^{i\theta_{1}}+e^{i\theta_{2}}) \\ i{(e}^{i\theta_{1}}+e^{i\theta_{2}}) & {-e}^{i\theta_{1}}+e^{i\theta_{2}} \end{matrix} \right]$$

Expanding one of these terms we see that:

$$e^{i\theta_{1}}-e^{i\theta_{2}}= Cos\left( \theta_{1} \right)-Cos\left( \theta_{2} \right)+i\left( Sin\left( \theta_{1} \right)+Sin\left( \theta_{2} \right) \right)= -2 Sin\left( \frac{\theta_{1}+\theta_{2}}{2} \right)Sin\left( \frac{\theta_{1}-\theta_{2}}{2} \right)+2iSin\left( \frac{\theta_{1}-\theta_{2}}{2} \right)Cos\left( \frac{\theta_{1}+\theta_{2}}{2} \right)$$

Simplifying this expression:

$$T_{11}= 2Sin\left( \Delta\right)\left( -Sin\left( \phi\right)+i Cos\left( \phi\right) \right)=2Sin\left( \Delta\right)*ie^{i\phi}$$

$$\phi=\frac{\theta_{1}+\theta_{2}}{2} \Delta= \frac{\theta_{1}-\theta_{2}}{2}$$

Expanding the rest of the terms we get the expression in the main text:

$$T\left( \theta_{1},\theta_{2} \right)= {ie}^{i\phi}\left[ \begin{matrix} sin(\Delta) & \cos\left( \Delta\right) \\ \cos\left( \Delta\right) & -sin(\Delta) \end{matrix} \right]$$

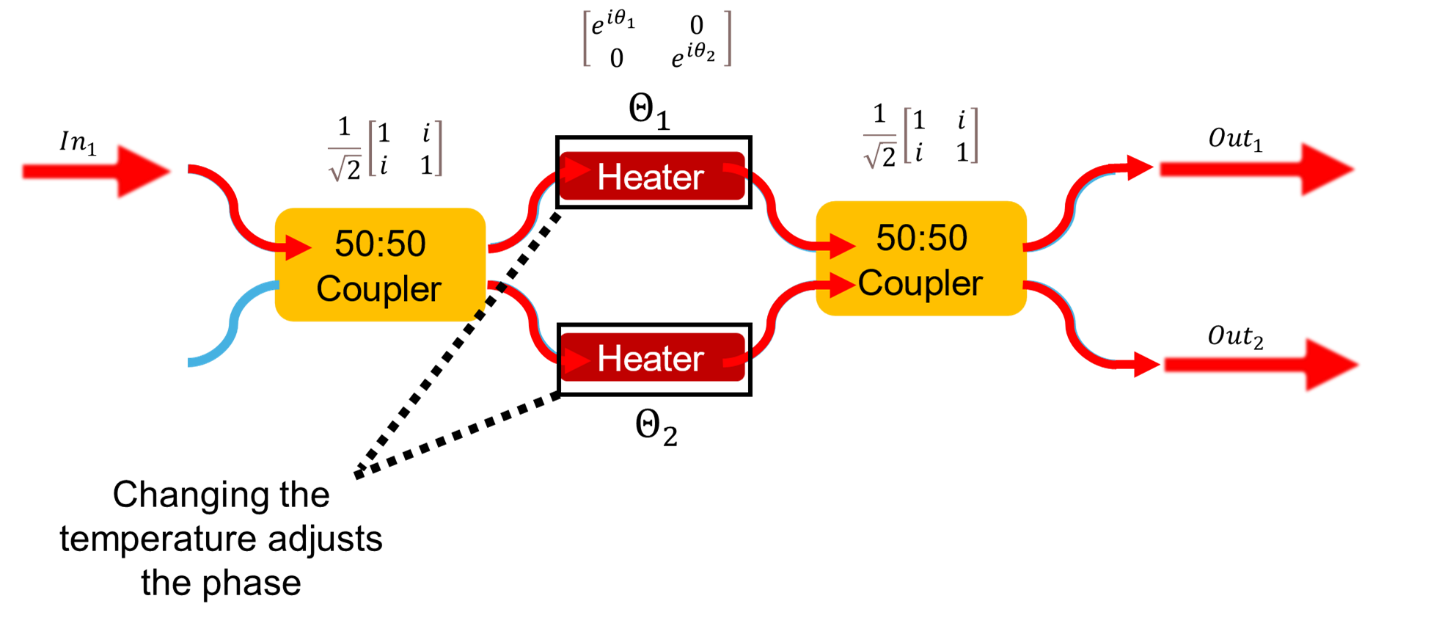
While the main work here is entirely simulation, it is a larger version of a device that currently exists. The typical losses of the device are as follows: 0.5dB loss per PUC, 2-3dB coupling loss to chip and 6-8dB loss routing through the chip to the desired MZI. Further details can be found on the supplementary note of Ref. [3]

Figure S2: Detailed description of how the light can move through the PUC. Depending on the values of these thermal phase shifters, the light can be programmed to move in any combination of output 1 and 2. This is how the ring resonators are programmed in the hexagonal pattern, by adjusting the heaters such that the light, incoming on port 1 exits only on output port 1. The same is done for 5 of 6 edges of the hexagon and the 6^th^ being the input and output port.

**Supplementary Note 3: Phase Shifter Progression**

To visualize the effect that changing the phase shifter has on the lattice, the phase shifter was swept from 0 to 2π in increments of 0.2π. The phase shifter center value is set to π and is what is shown in the main text. As the value of the phase shifter approaches 0/2π, the resonances of the link and site resonators begin to overlap. In this regime we are no longer implementing the quantum spin Hamiltonian. By introducing this overlap, they become new elements in the tight binding Hamiltonian rather than acting as a transition between site resonators (value of 0 in the Hamiltonian).


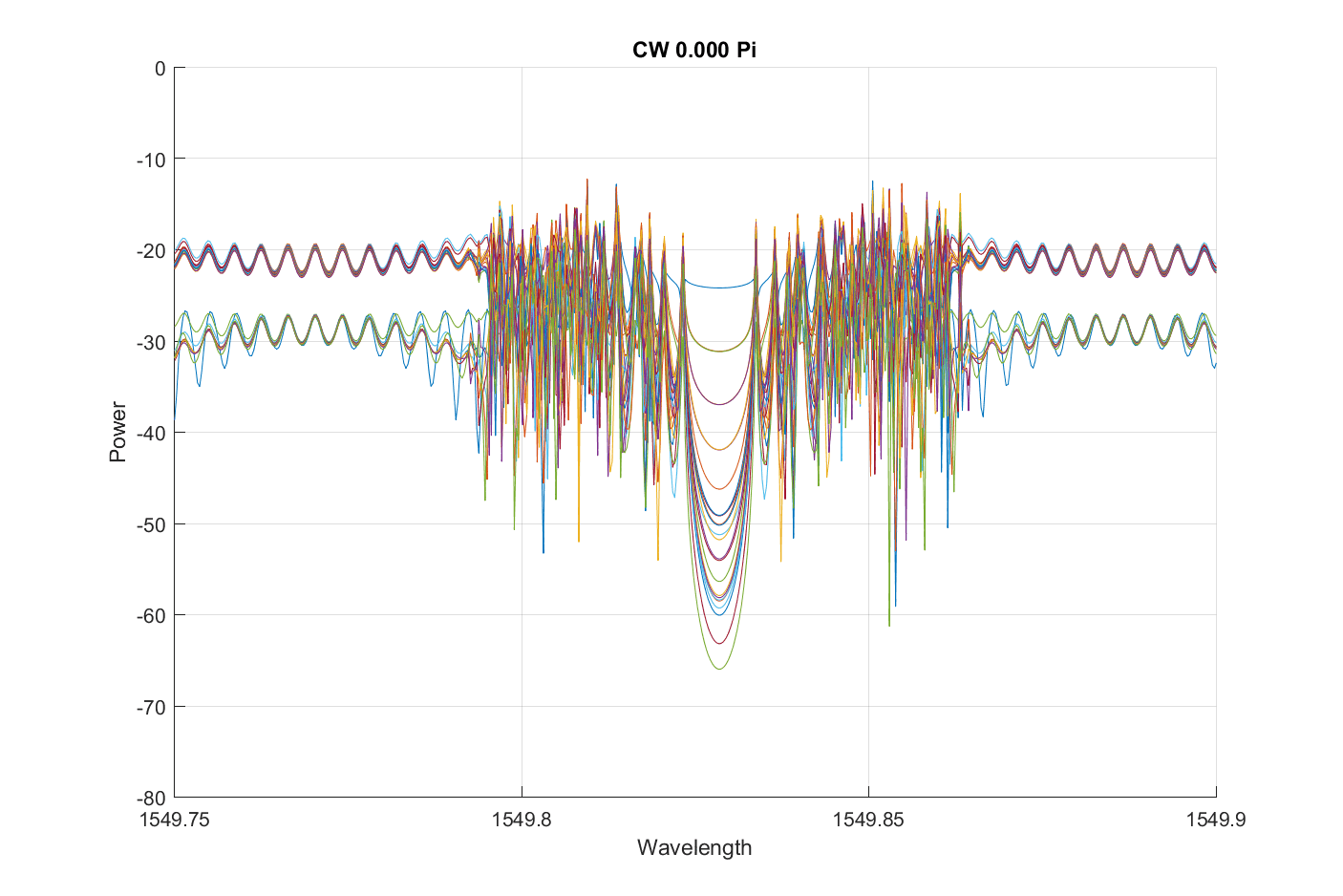


Figure S3: Sweeping the phase shifter uniformly from 0 to 2 π. At values of 0/2 π the system becomes another system as the link resonators become part of the hamiltonian.

**Supplementary Note 4: Sensitivity Analysis**

When random values are introduced on the phase shifters, it destroys the topological effects and misaligns the link resonances (Fig. S3a). This misalignment results in random overlap between the sites and links, leading to disorderly motion and eliminating the topological edges. In comparison, having uniform values on site resonators is essential to the integrity of the edge states where the resonant frequency of the sites and links, controlled by the phase shifter, is directly aligned with the spectral position of the topological states. Sweeping through the phase shifter values in unison cycles through the state’s FSR and at certain values the edges of two topological states overlap. As shown in Fig. S3b, once the phase shifters on the link sites are set to 0, i.e. the link and site resonators have the same resonant frequency, the edges of two states collide. The explanation for this is given in the previous supplementary note S3.

**
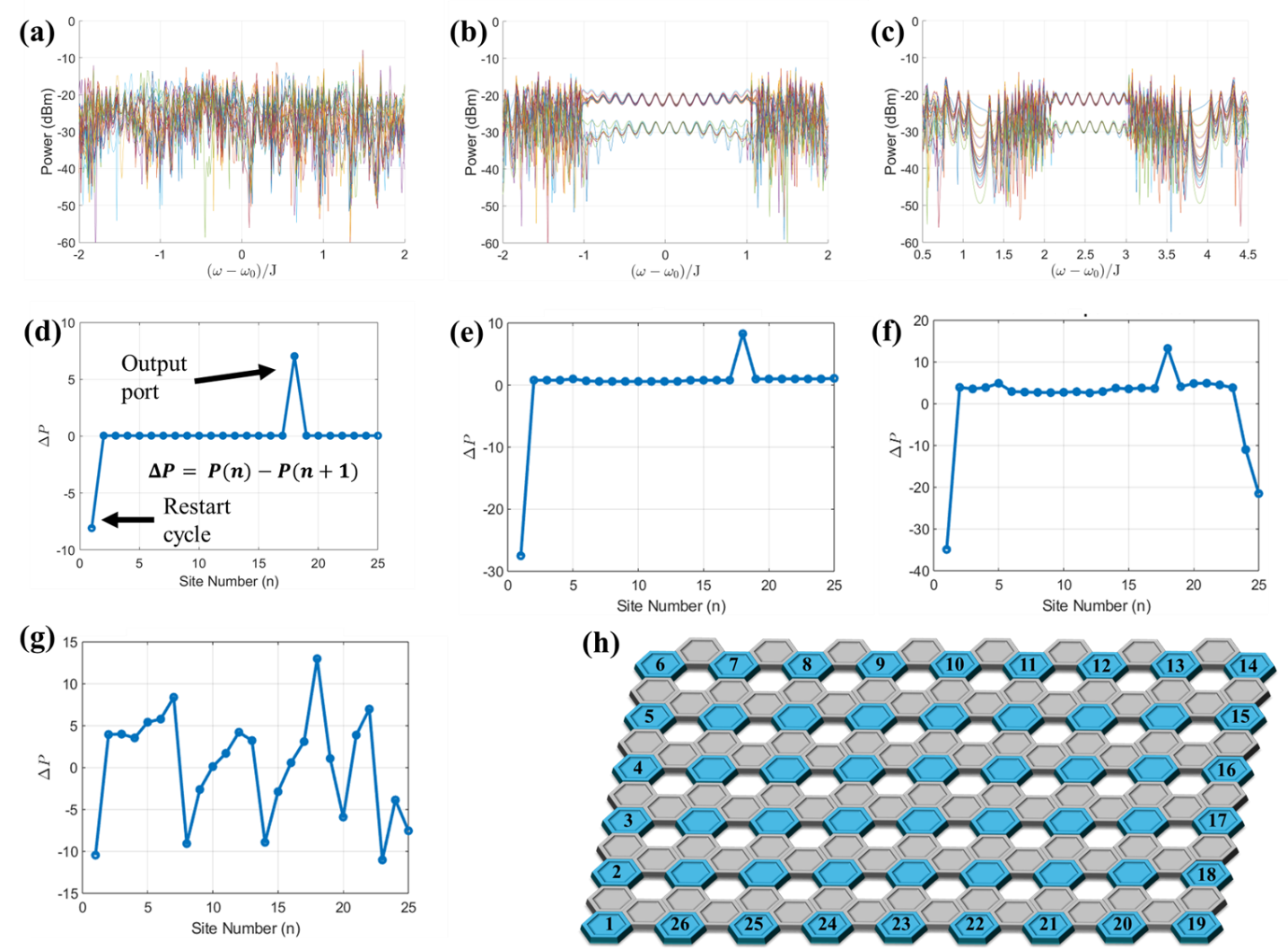
**

Figure S3: Raw spectral data with phase shifter changes. (a) Random (b) 0 phase shift on the link sites (c) π phase shift on the link sites. (d-g) the change in power from site n to site n+1. (d) Lossless case, (e) 0.1 dB loss per PUC, (f) 0.5 dB loss per PUC. On site 24 and 25, there is power that leaks from the resonator #1 into 26 and 25, hence the dip in $\Delta P$ at n=24/25. (g) Bulk state, 0.1 dB loss per PUC. The randomness of the bulk state causes large variation in the power difference. (h) Numbering system of lattice for reference.

In all the previous configurations, we had only considered the lossless case; however, the smaller fabricated device from iPronics exhibits approximately 0.5 dB loss per PUC. To ascertain the efficacy of topological states compared to bulk states in preserving path coherence, the same power differentials between site n and site n+1 are used. Figure S3d-g show the results of the following cases: lossless, 0.1 dB loss, 0.5 dB loss +0.05 std and the bulk state with 0.1dB loss. The lossless case shows the most consistency as to be expected. The peak corresponds to the loss at the output coupling port and the dip is the restarting of the cycle (P(26)-P(1)). As depicted in Fig. S2e-f, as losses accumulate, the consistency of power differentials diminishes, while the general form and integrity of the state persist. Conversely, examination of one of the bulk states reveals negligible correlation between site n and n+1.

**References:**

[1] M. Hafezi, S. Mittal, J. Fan, A. Migdall, and J. M. Taylor, "Imaging topological edge states in silicon photonics," *Nature Photonics,* vol. 7, no. 12, pp. 1001-1005, 2013/12/01 2013, doi: 10.1038/nphoton.2013.274.

[2] L. Huang *et al.*, "Hyperbolic photonic topological insulators," *Nature Communications,* vol. 15, no. 1, p. 1647, 2024/02/22 2024, doi: 10.1038/s41467-024-46035-y.

[3] A. Hashemi, E. L. Pereira, H. Li, J. L. Lado, and A. Blanco-Redondo, "Observation of non-Hermitian topology from optical loss modulation," *arXiv,* 2024, doi: <https://doi.org/10.48550/arXiv.2411.08729>.

1. [↑](#footnote-ref-2)
2. [↑](#footnote-ref-3)
